# The Associations Between Alanine Aminotransferase and Other Biochemical Parameters in Lean PCOS

**DOI:** 10.1007/s43032-022-01030-w

**Published:** 2022-07-21

**Authors:** Cai Liu, Kai Liu, Xiao Zhao, Junhua Zhu, Yang Liu, Lina Hao, Yanyun Gao, Peng Liu

**Affiliations:** 1 Department of Infertility Clinic, Yulin City First Hospital, Yulin, China; 2 Department of Gynecology, Northwest Women and Children’s Hospital, Xi’an, China; 3 Department of Intensive Care Unit, Xi’an Jiaotong University Second Affiliated Hospital, Xi’an, China; 4 Department of Hand and Foot Surgery, Yulin City First Hospital, Yulin, China

**Keywords:** Polycystic ovary syndrome, Alanine aminotransferase, Total testosterone, Liver damage, Associations

## Abstract

To explore the associations of alanine aminotransferase in lean women of polycystic ovary syndrome (PCOS) with other biochemical parameters and the potential risk factors. This is a retrospective cohort study with lean PCOS (*n* = 91) and healthy controls (*n* = 45); we reviewed the electrical records and databases of the PCOS patients in our infertility clinic between January 2019 and September 2021; independent *t*-test, linear correlation analysis, and multiple linear regression were used to explore the associations. Higher levels of luteinizing hormone, total testosterone, thyroid stimulating hormone, platelet count, lymphocyte count, homocysteine, alanine aminotransferase (ALT), and uric acid were identified in lean PCOS patients, while follicle-stimulating hormone level was lower in in lean PCOS as expected (*P* < 0.05). Of note, the linear correlation showed that *BMI*, total testosterone, white blood cell count, lymphocyte count, aspartate aminotransferase, and uric acid were positively associated with alanine aminotransferase (*r* = 0.232, 0.318, 0.218, 0.388, 0.602, 0.353 respectively, *P* < 0.05). After multiple linear regression was performed, total testosterone and aspartate aminotransferase were independently and positively correlated with alanine aminotransferase in lean PCOS (*B* = 0.251, 0.605 respectively, *P* < 0.05). Higher level of ALT was identified in the lean PCOS. *BMI*, white blood cell count, lymphocyte count, aspartate aminotransferase, uric acid, and total testosterone were positively correlated with ALT in lean PCOS. Total testosterone and aspartate aminotransferase were independently and positively associated with ALT in lean PCOS after multiple linear regression. There might exist a potential risk of afflicting liver impairment for the lean PCOS women in the earlier period. Early examination and intervention might be necessary to prevent or delay the progression of the liver disease as soon as the diagnosis of PCOS.

## Introduction

Polycystic ovary syndrome (PCOS) is a common endocrine disorder and a leading cause of infertility, the prevalence ranges between 5 and 21% of the reproductive women depending on the different definitions and studies [1, 2]. Stein and Leventhal described it in 1935 initially, along with the development of reproductive medicine, evidences have increasingly demonstrated that PCOS is a polygenic, polyfactorial, inflammatory, and autoimmune disease [3]; the physiopathologic mechanism is complicated and still unclear until today. A growing number of studies [4–6] suggest that PCOS is closely correlated with cardiovascular diseases and metabolic syndrome, such as type 2 diabetes mellitus and non-alcoholic fatty liver disease (NAFLD). Since simple steatosis may be a benign course, but steatohepatitis can lead to cirrhosis or hepatocellular carcinoma eventually [7], greatly damaging woman’s health; therefore, it seems extremely crucial to prevent the progression of the liver disease in clinical practice. Alanine aminotransferase (ALT), as a sensitive indicator of liver function, is increasingly significant and widely used in clinical practice; it is often treated as an initial marker of hepatic impairment and liver inflammation, and more sensitive than aspartate aminotransferase (AST) [8]. Studies [8, 9] demonstrated that there was a higher level of ALT among the PCOS patients, mainly owing to the increased prevalence of obesity, hyperandrogenism, insulin resistance, and dyslipidemia of the PCOS. However, the correlation between ALT and obese PCOS was reported more often than the lean one; moreover, the current situation is that the liver function test is not recommended routinely unless the patient is overweight or obese. This may cause the lean patients miss the best opportunity for intervention, such as modify lifestyles and make regular checking to prevent or delay the progression of the disease. This is why we conduct this research to seek for the correlation of ALT with other biochemical parameters in lean PCOS and the potential risk factors, offering some clinical evidences to elucidate the intricate pathogenic mechanism further.

## Methods

### Study Population

We reviewed the electrical records and databases of the PCOS patients who came to Infertility Clinic of Yulin First Hospital (a tertiary hospital in Shaanxi province, China) between January 2019 and September 2021 for infertility, and the controls were also extracted from our clinic record who came for a pre-pregnancy check-up, and all the tests of the controls are within the normal level and their normal ovulatory functions were confirmed by vaginal sonography. Clinical and laboratory materials were collected from electronic medical records; we also made a call to some of them for gathering the necessary information. This study was approved by the institutional research ethics review board of Yulin First Hospital (2022–001). Given the retrospective nature of the work, no specific consent was required from the patients.

The criteria of PCOS was diagnosed according to Rotterdam criteria [10], oligo-/anovulation, clinical or biochemical hyperandrogenism, ultrasound diagnosis of polycystic ovary morphology (PCOM), defined as the presence of at least one ovary > 10 ml, or 12 or more antral follicles 2–9 mm in diameter. PCOS was diagnosed if only two of the three are present, and patients with other causes of hyperandrogenism (congenital adrenal hyperplasia, Cushing’s syndrome, and androgenic-secreting tumors) and ovulation dysfunction, such as functional hypothalamic amenorrhea (FHA), thyroid dysfunction, and hyperprolactinemia (HPRL), were excluded. Exclusion criteria also included patients with diabetes, hypertension, hepatitis, endometriosis, recurrent pregnancy loss, any acute or chronic inflammation of the whole body, the people treated with any medication in 3 months, and with smoking and alcoholism history.

### Variables

Body mass index (*BMI*) was calculated by weight in kilograms divided by the square of the height in meters (kg/m^2^), and the one whose *BMI* ≥ 25 kg/m^2^ were excluded. The requirement of age was 20–40 years. The hormonal blood test was performed during the of 2–4th day of the menstrual period, and the biochemical blood was asked for an overnight fasting. Finally, we identified 91 patients of lean PCOS women as the study population and 45 healthy women as the controls. We collected general characteristics, the biochemical markers: the luteinizing hormone (LH), follicle-stimulating hormone (FSH), total testosterone (T), prolactin (PRL), thyroid-stimulating hormone (TSH), white blood cell count (WBC), neutrophil count (NEUT), lymphocyte count (LYMPH), monocyte count (MONO), platelet count (PLT), fasting plasma glucose (FPG), homocysteine (Hcy), erythrocyte sedimentation rate (ESR), ALT, AST, uric acid (UA), creatinine, urea, and cystatin levels. Owing to a retrospective study, we failed to get the data of insulin and lipid for they were not routine ones for the lean PCOS in the past.

### Statistical Analysis

PSS 23.0 was applied for all of the analysis, comparison of continuous variables were tested with Independent t-test or Mann–Whitney test. Pearson correlation analyses were used to evaluate correlations between continuous variables. Comparisons between groups were performed using single-factor ANOVA or non-parametric tests, and multiple linear regression was used to analyze independent correlated factors; *P* < 0.05 was considered to indicate statistical significance.

## Results

### General and Biochemical Indexes of Lean PCOS Women Compared with Healthy Controls

The two groups are comparable in age and *BMI*. However, higher levels of LH (*t* = 6.166, *P* = 0.000), total testosterone (*Z* =  − 5.535, *P* = 0.000), TSH (*Z* =  − 2.130, *P* = 0.033), platelet count (*t* = 2.607, *P* = 0.010), lymphocyte count (*t* = 2.199, *P* = 0.029), homocysteine (*Z* =  − 3.517, *P* = 0.000), ALT (*Z* =  − 2.436, *P* = 0.015), and uric acid (*t* = 3.715, *P* = 0.000) were identified in lean PCOS; FSH level was significantly lower in in lean PCOS as expected (*t* =  − 2.199, *P* = 0.029). No statistical significance was identified in PRL, ESR, neutrophil count, monocyte count, FPG, AST, creatinine, urea, and cystatin levels between the lean PCOS and controls (Table 1).

**Table1:** General and biochemical indexes of lean PCOS compared with controls

	Lean PCOS	Controls	*P* value^a^
*n*	91	45	
Age (y)	29.46 ± 3.50	30.38 ± 2.976	.096
BMI (kg/m^2^)	22.22 ± 1.64	21.97 ± 1.45	.342
LH (mIU/ml)	11.28 ± 10.04	5.16 ± 1.99	.000
FSH (mIU/ml)	6.19 ± 1.65	6.83 ± 1.59	.029
T (ng/ml)	0.3(0.19–0.45)	0.15(0.09–0.23)	.000
PRL (ng/ml)	14.34 ± 5.56	16.11 ± 6.56	.094
TSH (uIU/ml)	2.68(1.94–3.74)	2.21(1.7–3.1)	.029
WBC(× 10^9^/L)	6.30 ± 1.53	6.22 ± 1.61	.760
PLT (× 10^9^/L)	252.25 ± 60.06	227.60 ± 49.63	.010
MONO/L (× 10^9^/L)	0.39 ± 0.10	0.40 ± .12	.371
LYMPH/L (× 10^9^/L)	2.16 ± .62	1.94 ± .53	.029
NEUT/L (× 10^9^/L)	3.65 ± 1.12	3.75 ± 1.29	.603
ESR (mm/h)	4.40 ± 3.62	4.93 ± 3.01	.390
Hcy (umol/L)	12.1(9.7–17.7)	10.25(8.1–12.3)	.000
FPG (mmol/L)	5.23 ± .37	5.22 ± .31	.942
ALT (U/L)	15(12.0–21.2)	12(10–15.7)	.0015
AST (U/L)	17.54 ± 4.13	17.47 ± 4.26	.930
AST/ALT	1.22 ± 0.55	1.35 ± 0.34	.130
UA (umol/L)	271.00 ± 61.89	228.63 ± 51.28	.000
Crea(umol/L)	51.51 ± 9.01	51.99 ± 8.99	.767
Urea(mmol/L)	4.15 ± .89	4.30 ± 1.12	.439
Cystatin(mg/L)	.68 ± .10	.67 ± .10	.566

Values are expressed as mean ± *SD* for normal distribution or median (IQR) for non-normal distribution

^a^Differences between two groups were analyzed by independent *T*-test or Mann–Whitney test

*BMI*, body mass index; *LH*, luteinizing hormone; *FSH*, follicle-stimulating hormone; *T*, total testosterone; *PRL*, prolactin; *TSH*, thyroid-stimulating hormone; *WBC*, white blood cell count; *NEUT*, neutrophil count; *LYMPH*, lymphocyte count; *MONO*, monocyte count; *PLT*, platelet count; *FPG*, fasting plasma glucose; *Hcy*, homocysteine; *ESR*, erythrocyte sedimentation rate; *ALT*, alanine aminotransferase; *AST*, aspartate aminotransferase; *UA*, uric acid; *Crea*, creatinine

### Comparison of General and Biochemical Characteristics of Lean PCOS Women Between ALT Tertiles

**Fig. 1:**
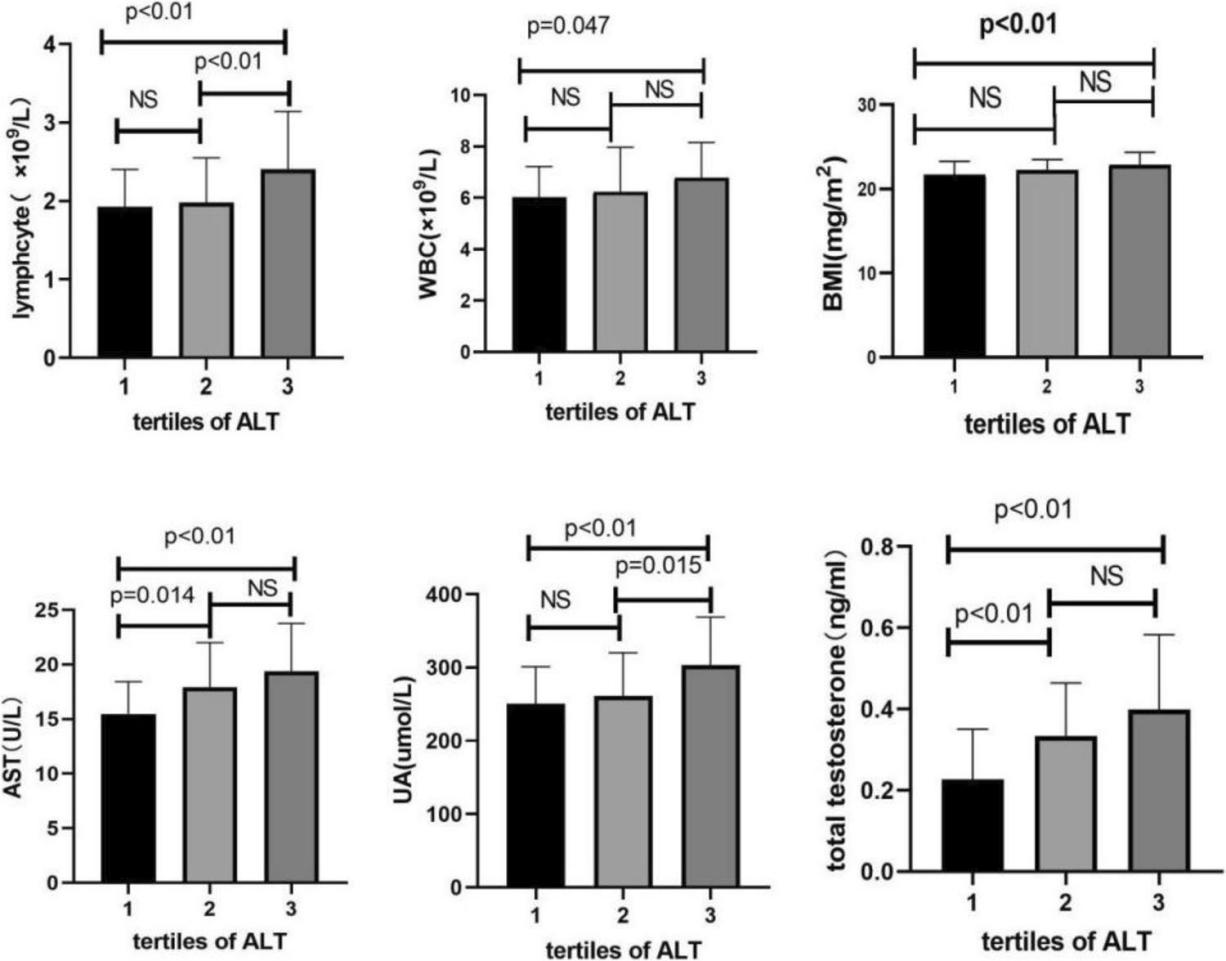
The specific differences of BMI, lymphocyte count, total testosterone, uric acid, AST, and white blood cell count between every two groups of the ALT tertiles in lean PCOS women. ALT, alanine aminotransferase; WBC, white blood cell count; BMI, body mass index; UA, uric acid; AST, aspartate aminotransferase; tertiles of ALT: 1, lower level of ALT group; 2, middle level of ALT group; 3, higher level of ALT group

We divided the lean PCOS patients into three subgroups based on the tertiles of ALT levels for the small sample, lower level of ALT (*n* = 31), middle level of ALT (*n* = 31), and higher level of ALT (*n* = 29). Of note, *BMI* (*F* = 4.8, *P* = 0.011), total testosterone (χ^2^ = 16.659, *P* = 0.000), lymphocyte count (*F* = 5.686, *P* = 0.0005), AST level (*F* = 7.903, *P* = 0.001), and UA (*F* = 45.138, *P* = 0.008) were significantly different; then, we compared the significant factors of every two groups one by one. The results illustrated a higher *BMI* (95%CI − 1.94 to − 1.95, *P* = 0.003) in the H-ALT than L-ALT level, but no statistical significance was identified between the other two groups (95%CI − 1.29 to 0.20, *P* = 0.152) (95%CI − 1.40 to 0.11, *P* = 0.097). Compared with the L-ALT group, higher total testosterone were showed in the M-ALT (95%CI − 0.18 to − 0.30, *P* = 0.007) and H-ALT (95%CI − 0.25 to − 0.94, *P* = 0.000), but no statistical significance between M-ALT and H-ALT groups (95%CI − 0.01 to 0.14, *P* = 0.101). AST in the M-ALT (95%CI − 4.38 to − 0.50, *P* = 0.014) and H-ALT (95%CI − 5.88 to − 1.92, *P* = 0.000) was remarkably higher than L-ALT level; however, there was no marked difference between the other two groups (95%CI − 3.42 to 0.52, *P* = 0.142). Higher level of UA was displayed in the H-ALT group than M-ALT (95%CI − 75.69 to − 8.46, *P* = 0.015) and L-ALT (95%CI − 87.69 to − 17.64, *P* = 0.004). Lymphocyte count was significantly higher in H-ALT group than L-ALT (95%CI − 0.79 to − 0.17, *P* = 0.003) and M-ALT group (95%CI − 0.73 to − 0.11, *P* = 0.008) and no significance between L-ALT and M-ALT group (95%CI − 0.24 to 0.36, *P* = 0.708). The white blood cell count was growing gradually from the L-ALT group to H-ALT; we also explored it between every two groups. Surprisingly, white blood count level in H-ALT was significantly higher than L-ALT group ((95%CI − 1.49 to − 0.01, *P* = 0.0047), but no statistical significance between the other two groups (95%CI − 0.94 to 0.54, *P* = 0.574) (95%CI − 1.29 to 0.20, *P* = 0.148) (Table 2, Fig. 1).

**Table 2:** Comparison of general and biochemical characteristics of lean PCOS between ALT tertiles

	L-ALT (group1)	M-ALT (group2)	H-ALT (group3)	*P* value^a^
*n*	31	31	29	
Age (y)	30.32 ± 3.59	29.77 ± 3.53	29.45 ± 3.45	.624
BMI (kg/m^2^)	21.67 ± 1.63	22.21 ± 1.29	22.86 ± 1.50	.011
LH (mIU/ml)	8.91 ± 5.56	12.24 ± 16.39	11.10 ± 6.44	.481
FSH (mIU/ml)	6.52 ± 1.83	6.16 ± 1.16	6.02 ± 1.77	.482
T (ng/ml)	0.2(0.16–0.27)	0.33(0.23–0.44)	0.39(0.22–0.57)	.000
PRL (ng/ml)	14.24 ± 5.70	14.51 ± 5.48	15.66 ± 5.71	.617
TSH (uIU/ml)	2.85 ± 1.06	2.76 ± 1.48	3.10 ± 1.39	.607
WBC(× 10^9^/L)	6.02 ± 1.19	6.23 ± 1.72	6.78 ± 1.37	.122
PLT (× 10^9^/L)	244.70 ± 63.67	242.29 ± 52.57	270.93 ± 58.98	.119
MONO/L (× 10^9^/L)	0.35 ± .09	0.39 ± .11	0.40 ± .09	.134
LYMPH/L (× 10^9^/L)	1.92 ± .48	1.97 ± .56	2.40 ± .73	.005
NEUT/L (× 10^9^/L)	3.59 ± 1.02	3.70 ± 1.42	3.84 ± .95	.721
ESR (mm/h)	4.68 ± 4.31	3.64 ± 2.45	5.12 ± 3.96	.319
HCY (umol/L)	13.97 ± 6.54	15.88 ± 11.06	13.50 ± 6.80	.565
FPG (mmol/L)	5.23 ± .27	5.20 ± .39	5.21 ± .42	.954
AST (U/L)	15.46 ± 2.95	17.91 ± 4.08	19.36 ± 4.39	.001
UA (umol/L)	250.46 ± 50.64	261.05 ± 59.00	303.13 ± 65.57	.008
Crea (umol/L)	51.93 ± 8.32	52.58 ± 9.04	49.85 ± 9.74	.498
Urea(mmol/L)	4.14 ± .88	4.23 ± .93	4.09 ± .89	.854
Cystatin(mg/L)	0.67 ± 0.93	0.68 ± .10	0.68 ± 0.10	.772

Values are expressed as mean ± *SD* for normal distribution or median (IQR) for non-normal distribution ^a^Differences between subgroups were analyzed by the single-factor ANOVA or non-parametric tests

*BMI*, body mass index; *LH*, luteinizing hormone; *FSH*, follicle-stimulating hormone; *T*, total testosterone; *PRL*, prolactin; *TSH*, thyroid-stimulating hormone; *WBC*, white blood cell count; *NEUT*, neutrophil count; *LYMPH*, lymphocyte count; *MONO*, monocyte count; *PLT*, platelet count; *FPG*, fasting plasma glucose; *Hcy*, homocysteine; *ESR*, erythrocyte sedimentation rate; *ALT*, alanine aminotransferase; *AST*, aspartate aminotransferase; *UA*, uric acid; *Crea*, creatinine

### Linear Correlation of ALT Level with the Hormone Indicators in Lean PCOS

As illustrated in Table 3, *BMI* and total testosterone were significantly and positively associated with ALT level (*r* = 0.232, 0.318 respectively, *P* < 0.05). No obvious linear association was identified in the level between ALT and LH, FSH, TSH, and PRL level (*P* > 0.05).

**Table 3:** The linear correlations of ALT with the hormone indicators in lean PCOS

	age (y)	BMI (kg/m^2^)	LH (mIU/ml)	FSH (mIU/ml)	TSH (uIU/ml)	T (ng/ml)	PRL (ng/ml)
ALT							
*r*	− 0.091	0.232	0.072	− 0.205	0.097	0.318	0.142
*P*^a^	0.393	0.027	0.509	0.059	0.373	0.003	0.195

^a^*P* value for test of significance of the associations using the Pearson correlation analysis. BMI, body mass index; LH, luteinizing hormone; FSH, follicle-stimulating hormone; T, total testosterone; PRL, prolactin; TSH, thyroid-stimulating hormone

### Linear Correlation of ALT Level with the Inflammatory Indicators in Lean PCOS

As illustrated in Table 4, white blood cell count and lymphocyte count were significantly and positively associated with ALT (*r* = 0.218,0.388 respectively, *P* < 0.05). No obvious linear association was identified in the level between ALT and neutrophil count, monocyte count, platelet count, ESR, and homocysteine level (*P* > 0.05).

**Table 4:** The linear correlations of ALT with the inflammatory indicators in lean PCOS

	WBC(× 10^9^/L）	NEUT(× 10^9^/L)	LIMPH(× 10^9^/L)	MONO(× 10^9^/L)	PLT(× 10^9^/L)	ESR (mm/h)	Hcy (umol/L)
ALT							
*r*	0.218	0.552	0.000	0.093	0.161	− 0.019	− 0.048
*P*^a^	0.038	0.063	0.388	0.383	0.128	0.865	0.676

^a^*P* value for test of significance of the association using the Pearson correlation analysis

*WBC*, white blood cell count; *NEUT*, neutrophil count; *LYMPH*, lymphocyte count; *MONO*, monocyte count; *PLT*, platelet count; *ESR*, erythrocyte sedimentation rate; *Hcy*, homocysteine

### Linear Correlation of ALT Level with the Metabolic Indexes in Lean PCOS

As illustrated in Table 5, AST and uric acid were positively and significantly associated with ALT level (*r* = 0.602, 0.353 respectively, *P* < 0.05). No obvious linear association was identified in the level between ALT and FPG, creatinine, urea, and cystatin level (*P* > 0.05).

**Table 5:** The linear correlations of ALT with the metabolic indexes in lean PCOS

	FPG (mmol/L)	UA (umol/L)	Crea (umol/L)	Urea(mmol/L	AST (U/L)	Cystatin(mg/L)
ALT						
r	0.061	0.353	-0.155	-0.036	0.602	0.049
P^a^	0.574	0.003	0.144	0.766	0.000	0.644

^a^*P* value for test of significance of the association using the Pearson correlation analysis

*ALT*, alanine aminotransferase; *FPG*, fasting plasma glucose; *UA*, uric acid; *AST*, aspartate aminotransferase; *Crea*, creatinine

### Multiple Linear Regression of Tertiles of ALT with the Metabolic Markers in Lean PCOS

Multiple linear regression was performed to analyze the independent correlations between ALT and other parameters if there was a statistically significant association with ALT in the univariate regression analysis or if it was clinically indicated. Total testosterone (*B* = 0.251, *P* < 0.01) and AST (*B* = 0.605, *P* < 0.01) were identified to be independently and positively correlated with ALT in lean PCOS. No significantly independent correlation was found in *BMI* (*B* = 0.113, *P* = 0219), uric acid (*B* = 0.160, *P* = 0.092), white blood count (*B* = 0.067, *P* = 0.088), and lymphocyte count (*B* = 0.174, *P* = 0.073) with ALT (Table 6).

**Table 6:** The multiple linear regression of ALT with the biochemical indexes in lean PCOS

Parameter	*B*	*t*	*P* value^a^
BMI (kg/m^2^)	0.113	1.242	0.219
WBC (× 10^9^/L)	0.067	0.712	0.088
Lymphocyte (× 10^9^/L)	0.174	1.825	0.073
AST (U/L)	0.605	6.679	0.000
T (ng/ml)	0.251	2.775	0.007
UA (umol/L)	0.160	1.71	0.092

^a^*P* value for test of significance of the association using the multiple linear correlation analysis

*BMI*, body mass index; *WBC*, white blood cell count; *AST*, aspartate aminotransferase; *T*, total testosterone; *UA*, uric acid

## Discussion

PCOS is a prevalent reproductive endocrine disorder, often accompanied by infertility, metabolic syndrome, and cardiovascular disease. Our report demonstrated consistently with previous researches [11] that LH is higher and FSH is lower in lean PCOS than the controls. Besides, despite we excluded the one with the thyroid dysfunction, TSH level was significantly higher in lean PCOS, revealing a similar result with other reports [12]. PCOS women are prone to have higher TSH levels owing to the disorder of hypothalamic-pituitary-ovarian axis (HPOA) in PCOS as we all know. Additionally, several studies [12, 13] suggest that higher TSH level is also correlated with metabolic syndrome in PCOS; Emel et al. [14] revealed that obese children demonstrate an increase in TSH levels as the degree of hepatic steatosis increased. Recently, another study [15] reported a strong link between TSH level and NAFLD proved by biopsy, independent of obesity, suggesting that thyroid hormone directly affect the synthesis and metabolism of cholesterol and fatty acids in an autonomous way by regulating the transcription of target genes involved in liver metabolism. However, our study failed to show statistical significance between ALT and TSH levels in lean PCOS, perhaps owing to the normal range of TSH levels in our study. This issue deserves to be explored further on account of its higher incidence in PCOS. In addition, PRL is negatively associated with AST, ALT, even after adjusting for age and *BMI* [16], indicating that lower serum PRL may damage liver cells, but the specific mechanism is unclear currently. However, there was no statistical significance showed in our study, and the reason behind this remains unknown, which needs to be studied in the future.

Furthermore, it is well-known that hyperandrogenism is a principal feature of PCOS, excessive production of androgen is the leading cause of the PCOS [17]. Our study demonstrated congruently with the common view that total testosterone is higher than controls in lean PCOS women. What is widespread acknowledged is that hyperandrogenism plays a role in almost all the complications of PCOS, for example, hyperandrogenism is also implicated tightly in elevated level of uric acid [18], which often accompanied by metabolic disease and cardiovascular disease [19]. Total testosterone was the independent risk factor of ALT in lean PCOS in our study, in line with earlier study [20], mainly because androgen can adversely affect mitochondrial function of liver cells, cause the imbalance between apoptosis and autophagy, resulting in liver damage [21]. It also affects the pathway of branched chain amino acid and the degradation of related mitochondrial enzymes, aggravating liver injury [22]. Therefore, PCOS with higher androgen are more predisposed to liver damage, which should be paid more attention in practice, regardless of their weight.

Simultaneously, homocysteine in the lean PCOS was also significantly higher than the healthy controls; this is beneficial in corroborating the higher risk of cardiovascular disease in PCOS for the people with higher homocysteine incline to get microthrombus in the vessels [23], and considered to be an independent risk factor for atherogenic and thrombotic components of various systems [24]. Besides, elevated homocysteine level is also tightly linked with fatty liver and chronic kidney disease [25, 26], while others [27] observed the opposite; they [27, 28] supposed the homocysteine levels are more higher in severe liver disease, but not in the mild one. According to a recent report [29], consensus on this issue has not reached yet, and the mechanism remains unknown. However, no significant correlation between the homocysteine levels with ALT was found in lean PCOS here. Since PCOS women seem to show more higher levels of homocysteine, the specific mechanism and effects on liver disease should be performed further by a well-designed and prospective study to clarify the associations between them.

In this study, the platelet count and lymphocyte count are remarkably higher in lean PCOS than healthy controls, supported by other researches [30–32]; it may be expounded by the mechanism of chronic inflammation in PCOS [3, 33], since the inflammatory state of PCOS may trigger an increased platelet count, but the higher platelet does not correlate with the inflammation markers [32]; therefore, the preexisting procoagulant state in PCOS might be caused by platelet activation and endothelial dysfunction [34]. However, our study failed to demonstrate marked differences in white blood cell count, neutrophil count, monocyte count, and erythrocyte sedimentation rate. Of interest, when we divided the lean PCOS into three subgroups and found that white blood cell count, lymphocyte count is positively associated with ALT levels in the lean PCOS. As mentioned above, being inflammation markers, perhaps white blood cell count and lymphocyte count also play roles in the higher levels of ALT in lean PCOS. Mounts of evidences [3, 35] suggest that PCOS is a state of chronic low-grade inflammation; immune system will activate while sensing the inflammatory factors. As a key metabolic organ, there might exist underlying inflammation in the liver cells in spite of the mild higher or normal range of ALT levels, leading to chronic liver damage.

ALT, a readily available, inexpensive, and routine metabolic marker used in clinical practice [36], has been observed elevated in various metabolic disorders, such as obesity, hyperlipidemia, and diabetes mellitus [37]. Even though our study population are lean ones and the ALT levels did not show clinical significance in practice, a remarkably statistical difference was also demonstrated in lean PCOS women. Being a good predictor of liver damage, ALT reflects more sensitively in variations of the liver [38], which reminds us of the liver injury may exist in the lean PCOS women in an earlier period during which we might ignore before, and they appear to be at higher risks in developing metabolic disease. Perhaps, we should also advise the lean PCOS women to make regular liver function checking and modify their lifestyles, such as making exercise, changing eating habits as early as possible to prevent the progression of liver disease; simultaneously, some researches were published on the reverse of the ALT level; for instance, Javed et al. [39] reported an improvement on the marker of liver injury and fibrosis through vitamin D supplementation in overweight and obese PCOS. Certainly, some better indicators and advice are still necessary for us to explore in the future. Additionally, uric acid was also statistically significant between the lean PCOS and controls in our study; however, there were no significant differences in FPG, creatinine, urea, cystatin, and AST levels. It is noting that people who have higher ALT levels tend to have higher uric acid and AST levels in our study. A study published lately demonstrated an independent and significant correlation between hyperuricemia and ALT level, even after adjusting for potential confounders [40], suggesting that insulin resistance, metabolic syndrome, and systemic inflammation might be caused by hyperuricemia, rather than a simple marker [40, 41], leading to steatohepatitis or even aggravating alcoholic or viral hepatitis [42]. Therefore, people with higher ALT level are more likely to develop severe metabolic disease and cardiovascular disease. However, uric acid level is not independently related with ALT level in our study after multiple factor analysis, which might be expounded by a mild metabolic matter in lean PCOS. AST, as another indicator of liver function, is independently correlated with ALT here, which may corroborate the fact that the combination has better sensitivity in clinical practice. Furthermore, our report also failed to show significant differences in FPG between subgroups, which is consistent with Belan M [43] rather than Chen MJ [8]; we speculate this may be related with the lean PCOS in our study population; for the elevated fasting, glucose is more linked with obesity, not PCOS [44]. Above all, ALT level is higher in lean PCOS women than healthy controls, affected by many metabolic parameters, and independently correlated with AST and total testosterone.

Our strength is that our data were truly from the infertility clinic, which represents the generality of lean PCOS patients and the laboratory tests were all from the same lab of our hospital; we excluded the one who had their partial tests out of our clinic, which also contributed to the small sample size. In addition, to our knowledge, this is one of the few studies to focus on the correlations of ALT level with inflammation markers, hormonal indicators, and metabolic indexes together in lean PCOS. What matters most is that the liver damage in lean PCOS are usually be ignored in practice owing to their normal weight. We have to admit that our study is a small and retrospective sample; hence, the incomplete information is unavoidable, such as the relations of ALT in the specific classification of PCOS needs to explore further in the future; the data of insulin resistance and blood lipid were absent for the these were not routine examinations for the lean PCOS in our clinic in the past. Patients with fatty liver were also unable to be specified or excluded due to the lack of ultrasound examination. Therefore, some large, well-designed and prospective studies are extremely necessary to ascertain our findings.

## Conclusions

In our study, higher ALT level was identified in the lean PCOS women. *BMI*, white blood cell count, lymphocyte count, AST, uric acid, and total testosterone were positively correlated with ALT in lean PCOS. Total testosterone and AST were independently and positively associated with ALT in lean PCOS after multiple linear regression. Our report reminds us of the potential risk of afflicting liver damage for the lean PCOS in the early period. Early examination and intervention might be necessary to prevent or delay the progression of the liver disease as soon as the diagnosis of PCOS, regardless of their weight. Surely, this study is a small sample and restricted to Chinese Han women, and further study is necessary to ascertain our findings.
